# A second ortho­rhom­bic polymorph of 3-ferro­cenylacrylaldehyde

**DOI:** 10.1107/S2056989026008170

**Published:** 2026-08-14

**Authors:** Evgeniya R. Shelukho, Vladimir P. Zaytsev, Victor N. Khrustalev, Tuncer Hökelek, Alebel N. Belay, Khudayar I. Hasanov, Narmina A. Guliyeva

**Affiliations:** a RUDN University, 6 Miklukho-Maklaya St., Moscow 117198, Russian Federation; b Zelinsky Institute of Organic Chemistry of RAS, Leninsky Prospect 47, Moscow 119991, Russian Federation; c Hacettepe University, Department of Physics, 06800 Beytepe-Ankara, Türkiye; d Department of Chemistry, Bahir Dar University, PO Box 79, Bahir Dar, Ethiopia; e Scientific Research Centre (SRC), Azerbaijan Medical University, A. Kasumzade St. 14, AZ 1022, Baku, Azerbaijan; f Department of Chemical Engineering, Baku Engineering University, Hasan Aliyev str. 120, AZ0101, Khirdalan, Absheron, Azerbaijan; University of Aberdeen, United Kingdom

**Keywords:** ferrocene derivatives, crystal structure, weak inter­actions

## Abstract

The title compound (systematic name: 3-ferrocenylprop-2-en-1-one), [Fe(C_5_H_5_)(C_8_H_7_O)] (**I**), crystallizing in the space group *P*2_1_2_1_2_1_, with *a* = 5.77599 (11) Å, *b* = 7.38297 (13) Å and *c* = 24.4180 (4) Å consists of fused ferrocene and acrylaldehyde moieties. In the crystal, C—H⋯O hydrogen bonds link the mol­ecules into infinite chains propagating along the *a*-axis direction and C—H⋯π inter­actions help to consolidate the packing. Compound (**I**) is a polymorph of the previously reported form of ferrocenylacrylaldehyde [Imhof (2004 ▸). *Acta Cryst.* E**60**, m1234–m1236], which also crystallizes in space group *P*2_1_2_1_2_1_ with cell parameters *a* = 7.9192 (2) Å, *b* = 11.1648 (3) Å and *c* = 12.4204 (4) Å. The Hirshfeld surface analysis of (**I**) indicates that the most important contributions to the crystal packing are from H⋯ (58.9%), H⋯C/C⋯H (22.6%) and H⋯O/O⋯H (17.5%) contacts. Computational methods revealed a C—H⋯O hydrogen-bonding energy of −10.9 kJ mol^−1^.

## Abstract 2

The title compound is a second ortho­rhom­bic polymorph of Fe(C_5_H_5_)(C_8_H_7_O) and crystallizes in the same space group as the first polymorph.

## 1. Chemical context

Ferrocene is the trivial name for bis­(η^5^-cyclo­penta­dien­yl)iron(II), Fe(C_5_H_5_)_2_, a prototypical metallocene comprising an iron atom sandwiched between two parallel negatively charged cyclo­penta­dienyl rings, which was first discovered and characterized at the beginning of the 1950s almost simultaneously by two scientific groups independently of each other (Kealy & Pauson, 1951 ▸; Miller *et al.*, 1952 ▸). The discovery of ferrocene more than seventy years ago has significantly influenced chemical research and provided a key impetus for establishing and rapidly expanding organometallic chemistry, which has continued at a rapid pace until now (Štěpnička, 2022 ▸). Over years of systematic study, ferrocene has proven to be an extraordinarily flexible core structure, with demonstrated value in asymmetric catalysis (Cunningham *et al.*, 2020 ▸), functional materials (Woo *et al.*, 2021 ▸), and medicinal (Sharma *et al.*, 2025 ▸) and analytical chemistry as well as many other research fields. Its success is underpinned by a rare synergy of stability, synthetic versatility, well-defined steric bulk, and electrochemically responsive redox properties that are readily adjustable to target requirements. Recent studies have demonstrated that ferrocene core, functionalized with various organic mol­ecules linkers, can poses anti­cancer activity against breast cancer (Mbaba *et al.*, 2020 ▸; Ashgar *et al.*, 2022 ▸), against lung cancer (Mazur *et al.*, 2022 ▸) and against leukaemia cell lines such as acute myeloid leukaemia and chronic lymphocytic leukaemia, demonstrating significant cytotoxicity and growth inhibition (Daum *et al.*, 2025 ▸; Tsypysheva *et al.*, 2020 ▸). As part of our studies in this area, we now report the synthesis and structure of the title compound, Fe(C_5_H_5_)(C_8_H_7_O) (**I**). It is a polymorph of the structure reported by Imhof (2004 ▸) in the same space group with *a* = 7.9192 (2) Å, *b* = 11.1648 (3) Å and *c* = 12.4204 (4) Å [Cambridge Structural Database (Groom *et al.*, 2016 ▸) refcode YAFVUX], which was crystallized from diethyl ether solution.
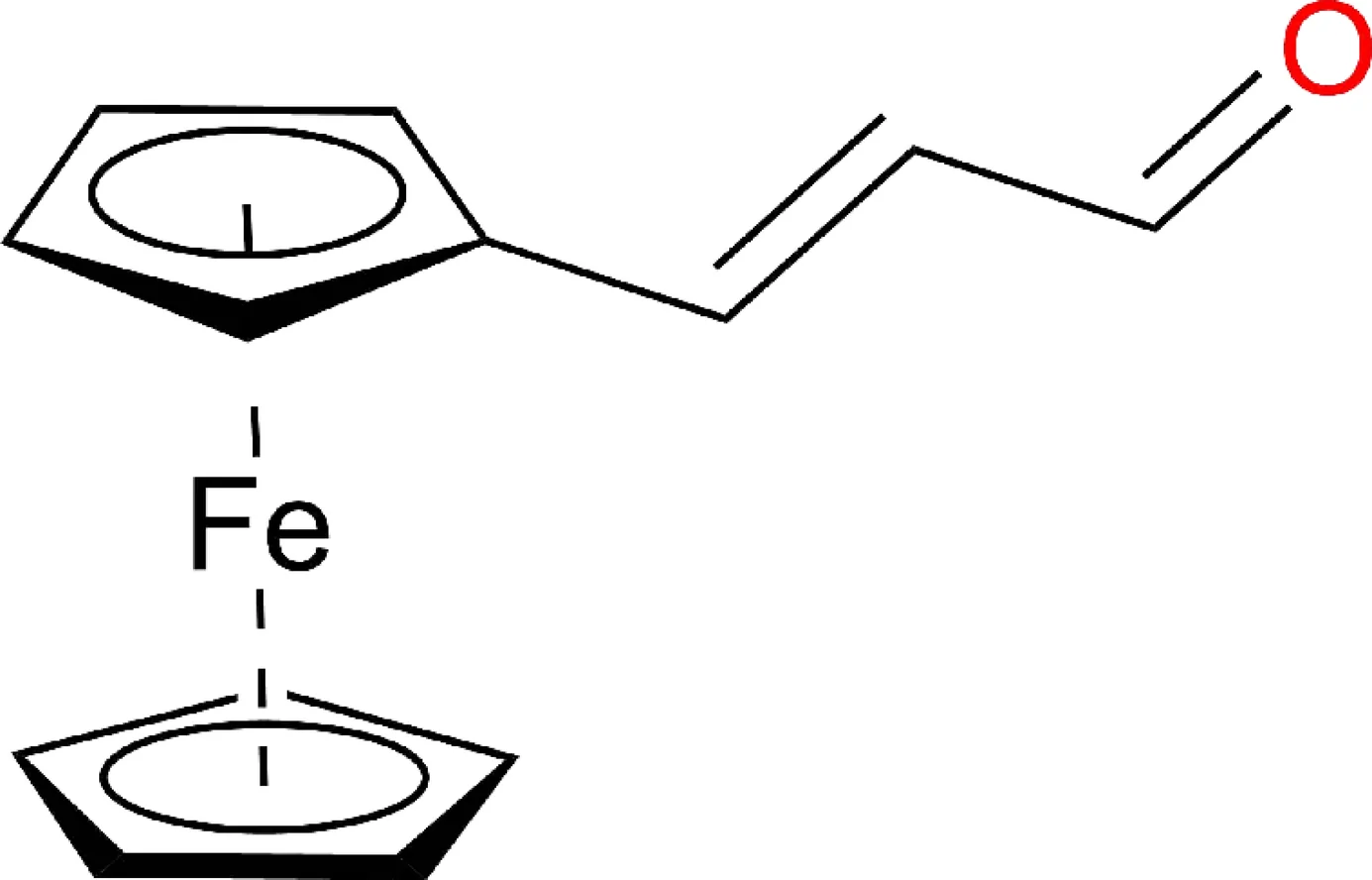


## 2. Structural commentary

**Figure 1:**
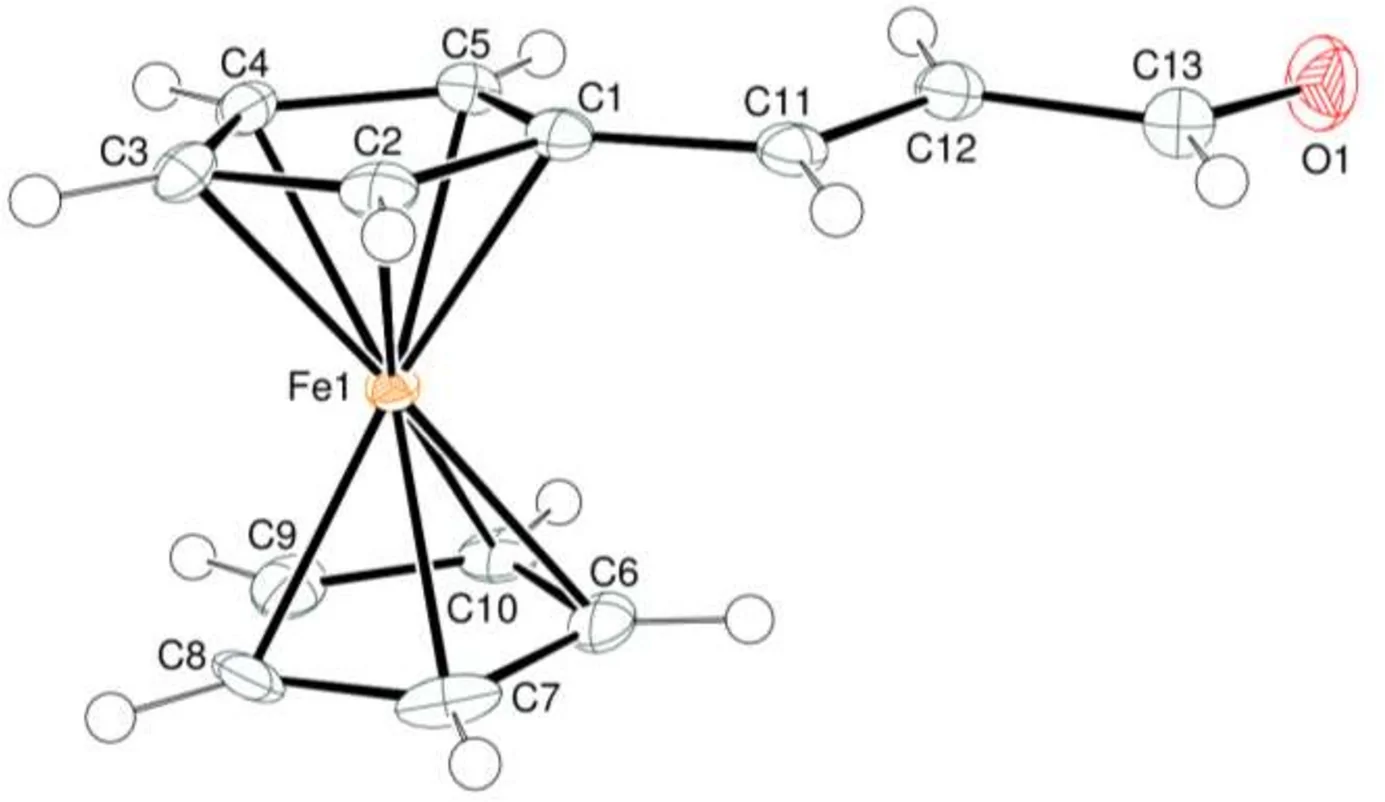
The mol­ecular structure of (**I**) with 50% probability ellipsoids.

Compound (**I**) consists of a ferrocene core with an attached acrylaldehyde group (Fig. 1 ▸). The centroid–centroid distance between the C1–C5 (*Cg*1) and C6–C10 (*Cg*2) cyclo­penta­dienyl rings is 3.2990 (17) Å [dihedral angle = 1.84 (16)° and slippage = 0.066 Å], which are almost eclipsed. The iron atom is almost equidistant between the rings [Fe1⋯*Cg*1 = 1.6455 (12) Å; Fe1⋯*Cg*2 = 1.6536 (14) Å; *Cg*1⋯Fe1⋯*Cg*2 = 178.40 (7)°]. The acrylaldehyde side chain is almost planar (r.m.s. deviation = 0.022 Å), where the O1—C13—C12—C11 and C1—C11—C12—C13 torsion angles are 176.8 (3) and −177.7 (2)°, respectively. The O atom of the aldehyde moiety is in an *s-trans* conformation with respect to the central C12—C13 single bond, and the C11=C12 [1.341 (4) Å] double bond is slightly elongated with respect to a typical C=C double bond length. The double [C1=O13 = 1.224 (4) Å and C11=C12] bonds lie on opposite sides of the central single [C12—C13 = 1.452 (4) Å] bond, forming a zigzag orientation, presumably minimizing steric hindrance. The C11—C12—C13 [120.2 (3)°] and C2—C1—C11 [124.7 (3)°] bond angles are significantly narrowed while the C1—C11—C12 [126.8 (3)°] and C5—C1—C11 [127.7 (2)°] bond angles are significantly enlarged with respect to the corresponding values [121.34 (16), 125.61 (16), 125.46 (16) and 126.76 (14)°, respectively] in the first polymorph of 3-ferrocenylprop-2-enal (Imhof, 2004 ▸).

## 3. Supra­molecular features

**Figure 2:**
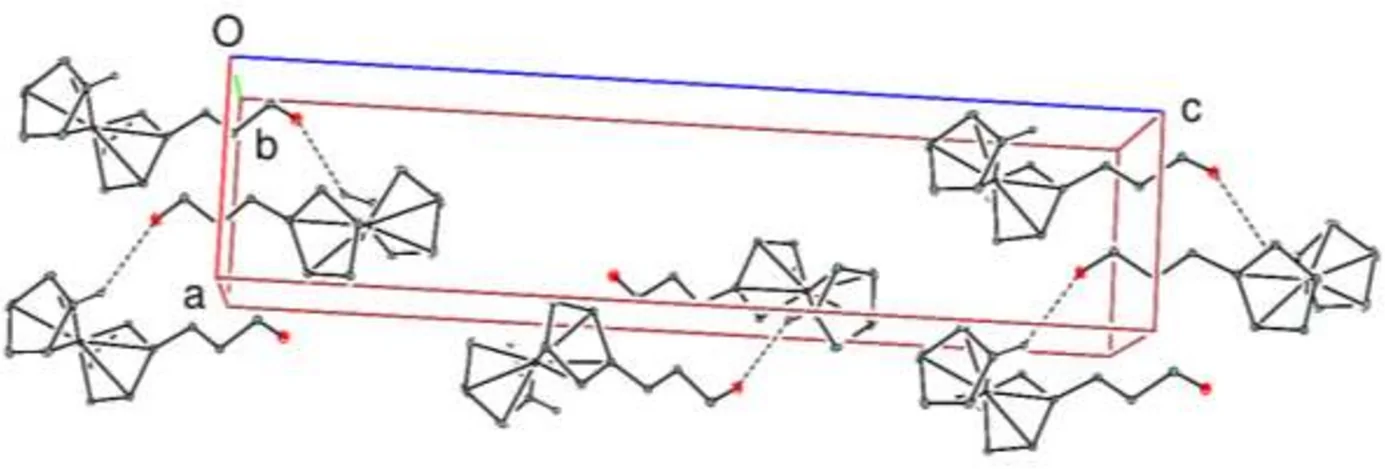
A partial packing diagram of (**I**) viewed down the *b*-axis direction with C—H⋯O hydrogen bonds shown as dashed lines. H atoms not involved in hydrogen bonds are omitted for clarity.

In the extended structure of (**I**), C—H⋯O hydrogen bonds (Table 1 ▸) link the mol­ecules into infinite chains propagating along the *a*-axis direction (Fig. 2 ▸) and weak C—H⋯π inter­actions help to consolidate the crystal packing.

**Figure 3:**
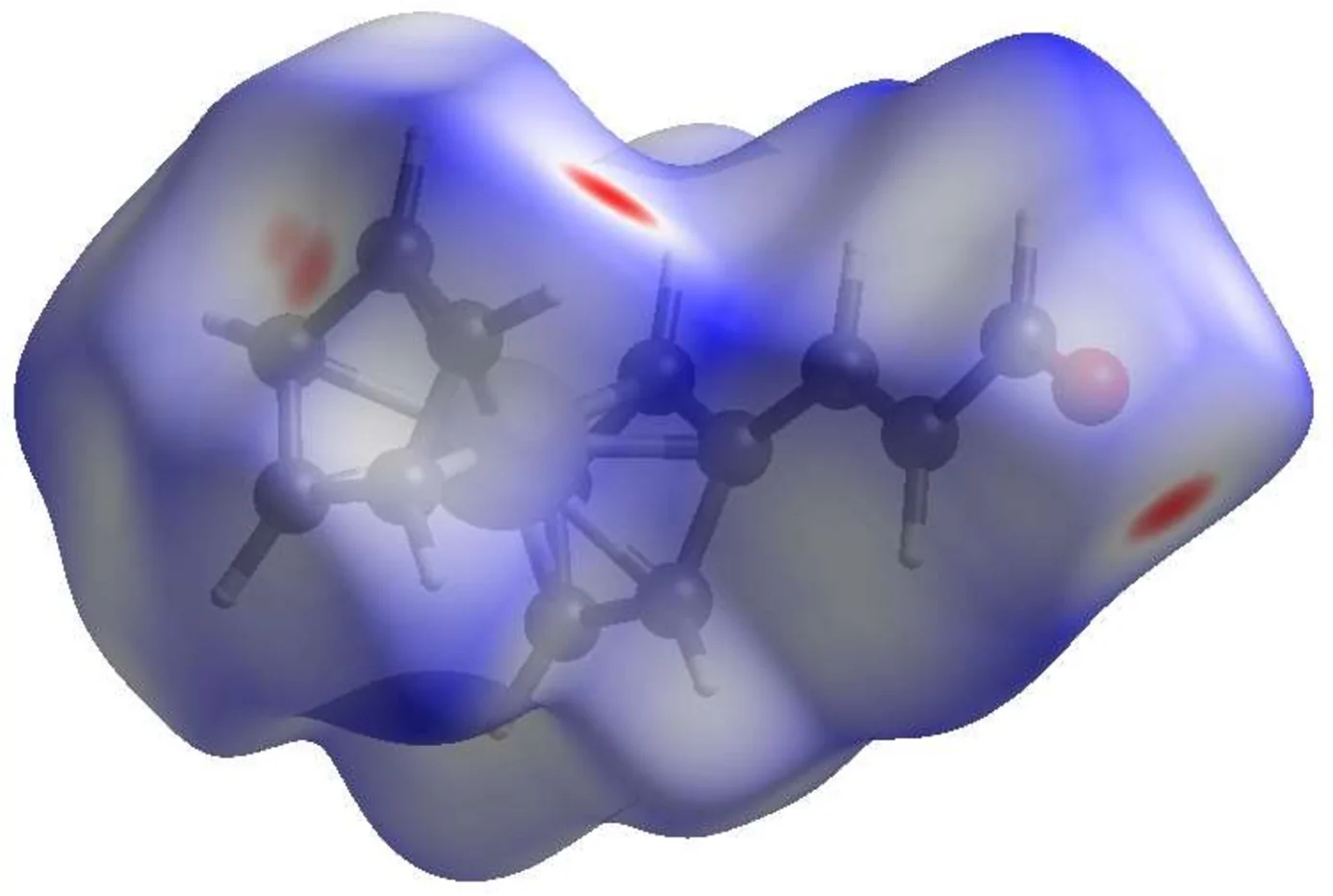
View of the three-dimensional Hirshfeld surface for (**I**) plotted over *d*_norm_ in the range −0.18 to 1.25 a.u.

**Figure 4:**
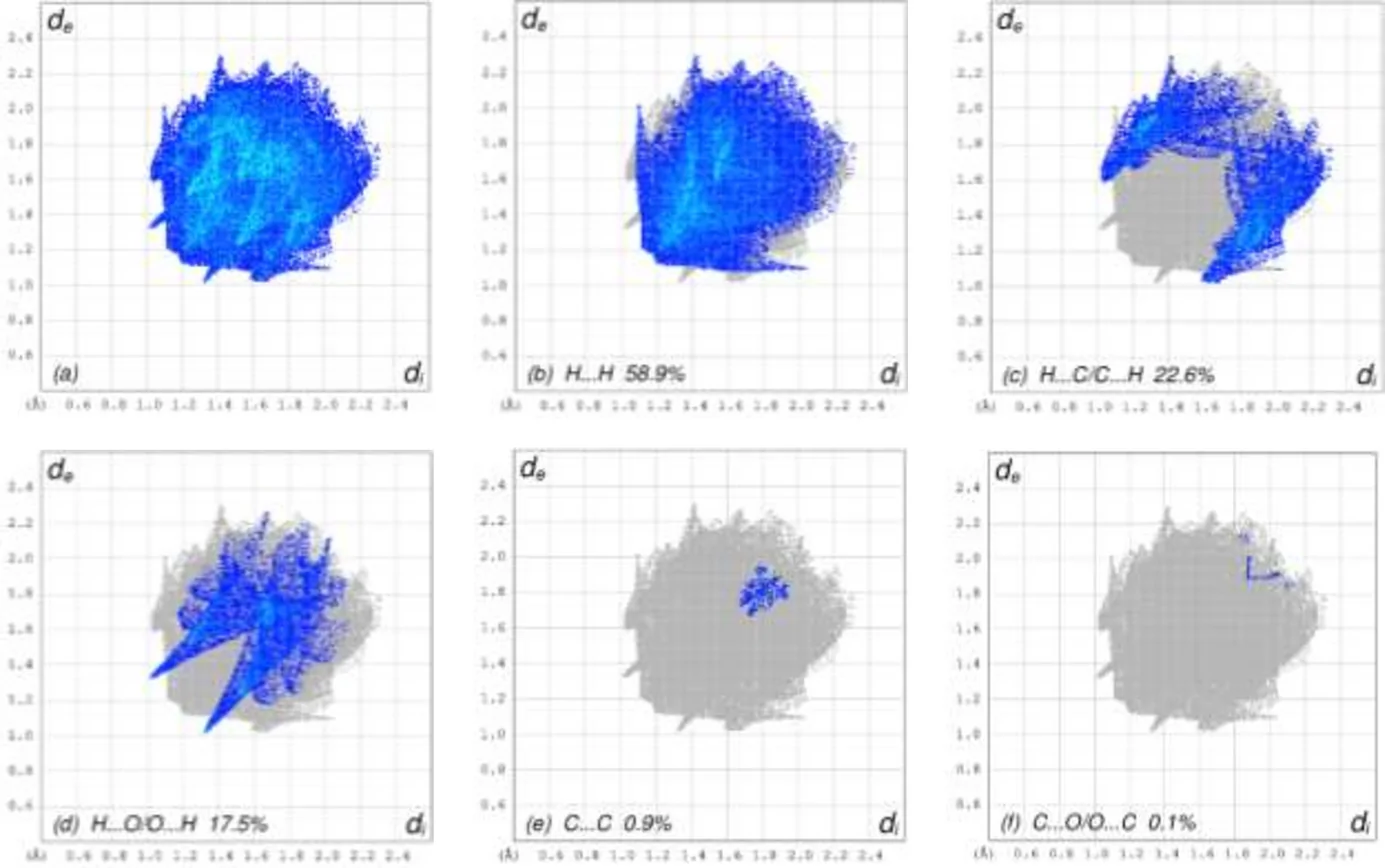
The two-dimensional fingerprint plots for (**I**), showing (*a*) all inter­actions, and delineated into (*b*)–(*f*) the various contact types.

The inter­molecular inter­actions in the crystal were visualized by carrying out a Hirshfeld surface (HS) analysis using *CrystalExplorer 17.5* (Spackman *et al.*, 2021 ▸). Fig. 3 ▸ shows the Hirshfeld surface mapped over *d*_norm_. The red spots indicate their roles as the respective donors and/or acceptors atoms in hydrogen bonding, as discussed above. The overall two-dimensional fingerprint plot is shown in Fig. 4 ▸*a* and those delineated into the different contact types are illustrated in Fig. 4 ▸*b*–*f*. According to the fingerprint plots, the H⋯H, H⋯C/C⋯H and H⋯O/O⋯H contacts make the most significant contributions to the HS, at 58.9%, 22.6% and 17.5%, respectively (Fig. 6).

A void analysis was performed to check the cohesion of the crystal. The volume of the crystal voids and the percentage of free space in the unit cell of (**I**) are 79.1 Å^3^ and 7.6%, respectively. These values compare with 126.0 Å^3^ and 11.5% in YAFVUX, which suggests that (**I**) is the more stable polymorph. This is supported by the difference in unit-cell volumes [1041.28 (3) Å for (**I**) and 1098.17 (5) Å for YAFVUX], although it should be note that the intensity data for (**I**) were collected at 100 K and those for YAFVUX at 183 K.

The inter­molecular inter­action energies were calculated using the CE–B3LYP/6–31G(d,p) energy model available in *CrystalExplorer 17.5* (Spackman *et al.*, 2021 ▸), where a cluster of mol­ecules is generated by applying crystallographic symmetry operations with respect to a selected central mol­ecule within the radius of 3.8 Å by default. The total inter­molecular energy (*E*_tot_) is the sum of electrostatic (*E*_ele_), polarization (*E*_pol_), dispersion (*E*_dis_) and exchange-repulsion (*E*_rep_) energies (Turner *et al.*, 2015 ▸) with scale factors of 1.057, 0.740, 0.871 and 0.618, respectively (Mackenzie *et al.*, 2017 ▸). Inter­action energies (in kJ mol^−1^) for (**I**) were calculated to be −2.0 (*E*_ele_), −0.7 (*E*_pol_), −16.2 (*E*_dis_), 9.4 (*E*_rep_) and −10.9 (*E*_tot_) for the C6—H6⋯O1 hydrogen bond.

## 4. Synthesis and crystallization

**Figure 5:**
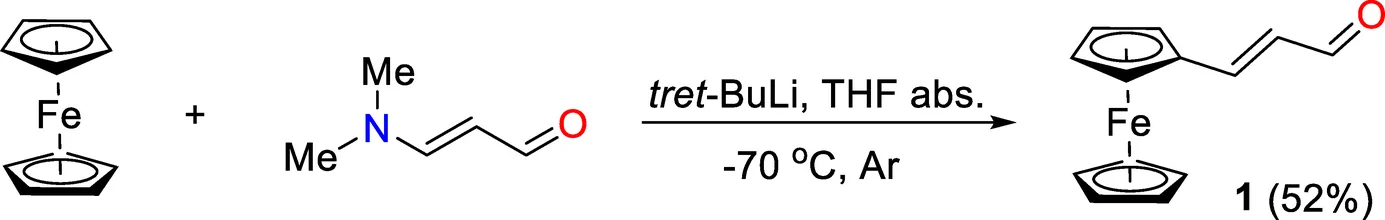
Synthesis scheme for (**I**).

Ferrocene (1.86 g, 10 mmol) was dissolved in tetra­hydro­furan (THF) (30 ml) and cooled to 203 K under an argon atmos­phere. A solution of *tert*-BuLi in pentane (17 mmol, 10 ml) was added to the reaction mixture dropwise under an Ar atmosphere and was stirred for 30 min. Then, 3-(di­methyl­amino)­acrolein (2.0 ml, 20 mmol) was added dropwise (Fig. 5 ▸). After that, the mixture was diluted to 10% aq. solution HCl, stirred at 278 K for 30 min and extracted with di­chloro­methane (DCM) (3 × 30 ml). The combined organic layers were dried over anhydrous Na_2_SO_4_ and filtered. The organic solvent was evaporated and the target product was purified by column chromatography (SiO_2_, 20 × 1.5 cm, eluent: EtOAc/hexane = 1/20); yield 52%, 1.248 g (5.2 mmol), m.p. 363–364 K. Single crystals of (**I**) in the form of red plates were grown from a solvent mixture of ethyl acetate and hexane at room temperature. ^1^H NMR (600.2 MHz, CDCl_3_) (*J*, Hz): δ 9.55 (*d*, *J* = 8.1 Hz, 1 H, CHO), 7.42 (*d*, *J* = 15.6 Hz, 1H, CH=CH), 6.34 (*dd*, *J* = 15.6, 8.1 Hz, 1 H, CH=CH), 4.55–4.51 (*m*, 4 H, H-Cp), 4.17 (*br. s*, 5 H, H-Cp). ^13^C NMR (150.9 MHz, CDCl_3_): δ 193.3, 155.2, 126.5, 77.9, 72.0 (2C), 70.1 (5C), 69.3 (2C). IR (KBr, cm^−1^): 1670 (C=O), 1618 (C=C).

## 5. Refinement

Crystal data, data collection and structure refinement details are summarized in Table 2 ▸. The hydrogen atom positions were calculated geometrically at distances of 0.95 Å and refined using a riding model with *U*_iso_(H) = 1.2*U*_eq_(C).

## Supplementary Material

Crystal structure: contains datablock(s) I, global. DOI: 10.1107/S2056989026008170/hb8242sup1.cif

Structure factors: contains datablock(s) I. DOI: 10.1107/S2056989026008170/hb8242Isup2.hkl

CCDC reference: 2579562

Additional supporting information:  crystallographic information; 3D view; checkCIF report

## Figures and Tables

**Table 1:** Hydrogen-bond geometry (Å, °) *Cg*2 is the centroid of the C6–C10 ring.

*D*—H⋯*A*	*D*—H	H⋯*A*	*D*⋯*A*	*D*—H⋯*A*
C6—H6⋯O1^i^	0.95	2.46	3.252 (3)	141
C8—H8⋯*Cg*2^ii^	0.95	2.82	3.736 (3)	161

Symmetry codes: (i) 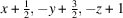; (ii) 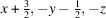.

**Table 2:** Experimental details

Crystal data	
Chemical formula	[Fe(C_5_H_5_)(C_8_H_7_O)]
*M*_r_	240.08
Crystal system, space group	Orthorhombic, *P*2_1_2_1_2_1_
Temperature (K)	100
*a*, *b*, *c* (Å)	5.77599 (11), 7.38297 (13), 24.4180 (4)
*V* (Å^3^)	1041.28 (3)
*Z*	4
Radiation type	Cu *K*α
μ (mm^−1^)	11.33
Crystal size (mm)	0.13 × 0.12 × 0.06

Data collection	
Diffractometer	XtaLAB Synergy, Single source at home/near, HyPix-Bantam
Absorption correction	Multi-scan (*CrysAlis PRO*; Rigaku OD, 2021 ▸)
*T*_min_, *T*_max_	0.235, 0.500
No. of measured, independent and observed [*I* > 2σ(*I*)] reflections	6966, 2068, 1988
*R*_int_	0.029
(sin θ/λ)_max_ (Å^−1^)	0.634

Refinement	
*R*[*F*^2^ > 2σ(*F*^2^)], *wR*(*F*^2^), *S*	0.026, 0.068, 1.07
No. of reflections	2068
No. of parameters	137
H-atom treatment	H-atom parameters constrained
Δρ_max_, Δρ_min_ (e Å^−3^)	0.33, −0.34
Absolute structure	Flack *x* determined using 691 quotients [(*I*^+^)−(*I*^−^)]/[(*I*^+^)+(*I*^−^)] (Parsons *et al.*, 2013 ▸)
Absolute structure parameter	0.008 (3)

Computer programs: *CrysAlis PRO* (Rigaku OD, 2021 ▸), *SHELXT* (Sheldrick, 2015*a* ▸), *SHELXL* (Sheldrick, 2015*b* ▸) and *SHELXTL* (Sheldrick, 2008 ▸).

## supplementary crystallographic information

### 3-Ferrocenylprop-2-en-1-one Crystal data

**Table:**

[Fe(C_5_H_5_)(C_8_H_7_O)]	*D*_x_ = 1.531 Mg m^−^^3^
*M**_r_* = 240.08	Cu *K*α radiation, λ = 1.54184 Å
Orthorhombic, *P*2_1_2_1_2_1_	Cell parameters from 4753 reflections
*a* = 5.77599 (11) Å	θ = 3.6–77.0°
*b* = 7.38297 (13) Å	µ = 11.33 mm^−^^1^
*c* = 24.4180 (4) Å	*T* = 100 K
*V* = 1041.28 (3) Å^3^	Plate, red
*Z* = 4	0.13 × 0.12 × 0.06 mm
*F*(000) = 496	

### 3-Ferrocenylprop-2-en-1-one Data collection

**Table:**

XtaLAB Synergy, Single source at home/near, HyPix-Bantam diffractometer	1988 reflections with *I* > 2σ(*I*)
Radiation source: micro-focus sealed X-ray tube	*R*_int_ = 0.029
ω scans	θ_max_ = 77.7°, θ_min_ = 3.6°
Absorption correction: multi-scan (CrysAlisPro; Rigaku OD, 2021)	*h* = −7→7
*T*_min_ = 0.235, *T*_max_ = 0.500	*k* = −9→9
6966 measured reflections	*l* = −30→18
2068 independent reflections	

### 3-Ferrocenylprop-2-en-1-one Refinement

**Table:**

Refinement on *F*^2^	Hydrogen site location: inferred from neighbouring sites
Least-squares matrix: full	H-atom parameters constrained
*R*[*F*^2^ > 2σ(*F*^2^)] = 0.026	*w* = 1/[σ^2^(*F*_o_^2^) + (0.0399*P*)^2^ + 0.3106*P*] where *P* = (*F*_o_^2^ + 2*F*_c_^2^)/3
*wR*(*F*^2^) = 0.068	(Δ/σ)_max_ < 0.001
*S* = 1.07	Δρ_max_ = 0.33 e Å^−^^3^
2068 reflections	Δρ_min_ = −0.34 e Å^−^^3^
137 parameters	Extinction correction: SHELXL, Fc^*^=kFc[1+0.001xFc^2^λ^3^/sin(2θ)]^-1/4^
0 restraints	Extinction coefficient: 0.0018 (2)
Primary atom site location: dual	Absolute structure: Flack *x* determined using 691 quotients [(*I*^+^)-(*I*^-^)]/[(*I*^+^)+(*I*^-^)] (Parsons *et al.*, 2013)
Secondary atom site location: difference Fourier map	Absolute structure parameter: 0.008 (3)

### 3-Ferrocenylprop-2-en-1-one Special details

**Table:**

Geometry. All esds (except the esd in the dihedral angle between two l.s. planes) are estimated using the full covariance matrix. The cell esds are taken into account individually in the estimation of esds in distances, angles and torsion angles; correlations between esds in cell parameters are only used when they are defined by crystal symmetry. An approximate (isotropic) treatment of cell esds is used for estimating esds involving l.s. planes.

### 3-Ferrocenylprop-2-en-1-one Fractional atomic coordinates and isotropic or equivalent isotropic displacement parameters (Å^2^)

**Table:**

	*x*	*y*	*z*	*U*_iso_*/*U*_eq_	
Fe1	0.25332 (7)	0.37182 (5)	0.34479 (2)	0.01172 (14)	
O1	0.2819 (4)	0.7387 (3)	0.57138 (9)	0.0316 (5)	
C1	0.2447 (6)	0.2947 (3)	0.42514 (9)	0.0157 (5)	
C2	0.3681 (5)	0.1630 (3)	0.39272 (10)	0.0163 (5)	
H2	0.5245	0.1270	0.3978	0.020*	
C3	0.2161 (5)	0.0969 (3)	0.35218 (10)	0.0176 (5)	
H3	0.2533	0.0088	0.3252	0.021*	
C4	−0.0028 (5)	0.1839 (4)	0.35833 (10)	0.0161 (5)	
H4	−0.1366	0.1631	0.3366	0.019*	
C5	0.0149 (5)	0.3083 (4)	0.40315 (10)	0.0156 (5)	
H5	−0.1043	0.3860	0.4160	0.019*	
C6	0.3912 (5)	0.6273 (4)	0.34172 (11)	0.0201 (6)	
H6	0.4386	0.6984	0.3721	0.024*	
C7	0.5323 (5)	0.5044 (4)	0.31226 (13)	0.0254 (7)	
H7	0.6903	0.4782	0.3195	0.030*	
C8	0.3956 (6)	0.4274 (4)	0.26994 (12)	0.0292 (8)	
H8	0.4463	0.3408	0.2438	0.035*	
C9	0.1703 (6)	0.5024 (4)	0.27361 (11)	0.0258 (7)	
H9	0.0432	0.4750	0.2504	0.031*	
C10	0.1680 (5)	0.6261 (4)	0.31821 (12)	0.0218 (6)	
H10	0.0390	0.6957	0.3301	0.026*	
C11	0.3472 (5)	0.4031 (4)	0.46809 (10)	0.0167 (5)	
H11	0.5093	0.3902	0.4734	0.020*	
C12	0.2373 (6)	0.5198 (3)	0.50116 (10)	0.0180 (5)	
H12	0.0743	0.5344	0.4984	0.022*	
C13	0.3669 (5)	0.6241 (4)	0.54122 (11)	0.0222 (6)	
H13	0.5284	0.6018	0.5442	0.027*	

### 3-Ferrocenylprop-2-en-1-one Atomic displacement parameters (Å^2^)

**Table:**

	*U*^11^	*U*^22^	*U*^33^	*U*^12^	*U*^13^	*U*^23^
Fe1	0.0133 (2)	0.0100 (2)	0.01192 (19)	0.0000 (2)	0.00116 (18)	0.00123 (12)
O1	0.0332 (14)	0.0303 (11)	0.0311 (10)	−0.0015 (11)	0.0040 (10)	−0.0137 (9)
C1	0.0179 (12)	0.0148 (10)	0.0143 (10)	−0.0004 (14)	0.0018 (12)	0.0045 (9)
C2	0.0177 (12)	0.0120 (11)	0.0190 (11)	0.0029 (11)	0.0022 (10)	0.0069 (10)
C3	0.0240 (14)	0.0082 (10)	0.0205 (11)	−0.0009 (11)	0.0022 (11)	0.0019 (9)
C4	0.0166 (12)	0.0129 (11)	0.0189 (11)	−0.0031 (11)	0.0022 (10)	0.0005 (10)
C5	0.0152 (12)	0.0171 (13)	0.0146 (11)	−0.0012 (11)	0.0023 (10)	0.0025 (10)
C6	0.0279 (15)	0.0138 (12)	0.0186 (12)	−0.0049 (12)	−0.0016 (11)	0.0053 (12)
C7	0.0156 (14)	0.0220 (14)	0.0385 (16)	−0.0009 (11)	0.0060 (12)	0.0150 (13)
C8	0.050 (2)	0.0190 (15)	0.0189 (13)	−0.0055 (15)	0.0220 (14)	−0.0003 (12)
C9	0.0365 (18)	0.0252 (15)	0.0158 (13)	−0.0102 (13)	−0.0082 (11)	0.0107 (11)
C10	0.0251 (14)	0.0132 (12)	0.0272 (13)	0.0021 (12)	0.0052 (11)	0.0105 (12)
C11	0.0172 (12)	0.0190 (13)	0.0138 (11)	−0.0024 (11)	−0.0004 (10)	0.0057 (10)
C12	0.0186 (13)	0.0190 (11)	0.0164 (11)	−0.0013 (13)	0.0017 (12)	0.0027 (9)
C13	0.0248 (14)	0.0218 (14)	0.0199 (12)	−0.0032 (13)	0.0005 (11)	0.0003 (11)

### 3-Ferrocenylprop-2-en-1-one Geometric parameters (Å, º)

**Table:**

Fe1—C5	2.036 (3)	C4—C5	1.432 (4)
Fe1—C1	2.043 (2)	C4—H4	0.9500
Fe1—C9	2.045 (3)	C5—H5	0.9500
Fe1—C8	2.046 (3)	C6—C10	1.411 (4)
Fe1—C7	2.046 (3)	C6—C7	1.416 (4)
Fe1—C2	2.046 (3)	C6—H6	0.9500
Fe1—C10	2.047 (3)	C7—C8	1.419 (5)
Fe1—C6	2.049 (3)	C7—H7	0.9500
Fe1—C3	2.049 (2)	C8—C9	1.418 (5)
Fe1—C4	2.055 (3)	C8—H8	0.9500
O1—C13	1.224 (4)	C9—C10	1.421 (4)
C1—C5	1.435 (4)	C9—H9	0.9500
C1—C2	1.442 (4)	C10—H10	0.9500
C1—C11	1.446 (4)	C11—C12	1.341 (4)
C2—C3	1.411 (4)	C11—H11	0.9500
C2—H2	0.9500	C12—C13	1.452 (4)
C3—C4	1.426 (4)	C12—H12	0.9500
C3—H3	0.9500	C13—H13	0.9500
			
C5—Fe1—C1	41.19 (11)	C2—C3—C4	108.8 (2)
C5—Fe1—C9	123.02 (12)	C2—C3—Fe1	69.72 (15)
C1—Fe1—C9	160.35 (13)	C4—C3—Fe1	69.88 (15)
C5—Fe1—C8	160.59 (13)	C2—C3—H3	125.6
C1—Fe1—C8	157.44 (15)	C4—C3—H3	125.6
C9—Fe1—C8	40.56 (14)	Fe1—C3—H3	126.4
C5—Fe1—C7	156.14 (13)	C3—C4—C5	107.8 (2)
C1—Fe1—C7	121.69 (13)	C3—C4—Fe1	69.46 (15)
C9—Fe1—C7	68.22 (13)	C5—C4—Fe1	68.81 (16)
C8—Fe1—C7	40.60 (14)	C3—C4—H4	126.1
C5—Fe1—C2	69.22 (11)	C5—C4—H4	126.1
C1—Fe1—C2	41.31 (10)	Fe1—C4—H4	127.2
C9—Fe1—C2	156.58 (12)	C4—C5—C1	107.9 (2)
C8—Fe1—C2	122.14 (13)	C4—C5—Fe1	70.21 (15)
C7—Fe1—C2	109.12 (12)	C1—C5—Fe1	69.67 (14)
C5—Fe1—C10	105.69 (11)	C4—C5—H5	126.0
C1—Fe1—C10	123.64 (11)	C1—C5—H5	126.0
C9—Fe1—C10	40.65 (12)	Fe1—C5—H5	125.7
C8—Fe1—C10	68.26 (12)	C10—C6—C7	108.4 (3)
C7—Fe1—C10	68.14 (12)	C10—C6—Fe1	69.77 (17)
C2—Fe1—C10	161.92 (12)	C7—C6—Fe1	69.66 (17)
C5—Fe1—C6	120.03 (12)	C10—C6—H6	125.8
C1—Fe1—C6	107.52 (11)	C7—C6—H6	125.8
C9—Fe1—C6	68.03 (11)	Fe1—C6—H6	126.3
C8—Fe1—C6	68.08 (12)	C6—C7—C8	107.9 (3)
C7—Fe1—C6	40.47 (12)	C6—C7—Fe1	69.87 (17)
C2—Fe1—C6	126.07 (11)	C8—C7—Fe1	69.70 (17)
C10—Fe1—C6	40.31 (12)	C6—C7—H7	126.1
C5—Fe1—C3	68.85 (11)	C8—C7—H7	126.1
C1—Fe1—C3	68.72 (10)	Fe1—C7—H7	125.9
C9—Fe1—C3	121.15 (11)	C9—C8—C7	107.9 (2)
C8—Fe1—C3	108.62 (12)	C9—C8—Fe1	69.69 (16)
C7—Fe1—C3	126.21 (12)	C7—C8—Fe1	69.71 (16)
C2—Fe1—C3	40.29 (11)	C9—C8—H8	126.0
C10—Fe1—C3	155.68 (12)	C7—C8—H8	126.0
C6—Fe1—C3	162.95 (12)	Fe1—C8—H8	126.1
C5—Fe1—C4	40.98 (11)	C8—C9—C10	108.0 (3)
C1—Fe1—C4	68.90 (11)	C8—C9—Fe1	69.75 (16)
C9—Fe1—C4	106.63 (12)	C10—C9—Fe1	69.74 (16)
C8—Fe1—C4	124.63 (12)	C8—C9—H9	126.0
C7—Fe1—C4	162.27 (12)	C10—C9—H9	126.0
C2—Fe1—C4	68.44 (11)	Fe1—C9—H9	126.1
C10—Fe1—C4	119.80 (12)	C6—C10—C9	107.9 (3)
C6—Fe1—C4	155.13 (12)	C6—C10—Fe1	69.92 (17)
C3—Fe1—C4	40.67 (11)	C9—C10—Fe1	69.60 (16)
C5—C1—C2	107.4 (2)	C6—C10—H10	126.1
C5—C1—C11	127.7 (2)	C9—C10—H10	126.1
C2—C1—C11	124.7 (3)	Fe1—C10—H10	126.0
C5—C1—Fe1	69.14 (13)	C12—C11—C1	126.8 (3)
C2—C1—Fe1	69.44 (13)	C12—C11—H11	116.6
C11—C1—Fe1	122.16 (18)	C1—C11—H11	116.6
C3—C2—C1	108.1 (2)	C11—C12—C13	120.2 (3)
C3—C2—Fe1	69.98 (14)	C11—C12—H12	119.9
C1—C2—Fe1	69.25 (13)	C13—C12—H12	119.9
C3—C2—H2	125.9	O1—C13—C12	124.4 (3)
C1—C2—H2	125.9	O1—C13—H13	117.8
Fe1—C2—H2	126.4	C12—C13—H13	117.8
			
C5—C1—C2—C3	−0.4 (3)	Fe1—C6—C7—C8	59.6 (2)
C11—C1—C2—C3	−175.0 (2)	C10—C6—C7—Fe1	−59.22 (19)
Fe1—C1—C2—C3	−59.39 (18)	C6—C7—C8—C9	−0.3 (3)
C5—C1—C2—Fe1	58.95 (16)	Fe1—C7—C8—C9	59.43 (19)
C11—C1—C2—Fe1	−115.6 (2)	C6—C7—C8—Fe1	−59.7 (2)
C1—C2—C3—C4	−0.2 (3)	C7—C8—C9—C10	0.1 (3)
Fe1—C2—C3—C4	−59.14 (18)	Fe1—C8—C9—C10	59.51 (18)
C1—C2—C3—Fe1	58.94 (17)	C7—C8—C9—Fe1	−59.44 (19)
C2—C3—C4—C5	0.8 (3)	C7—C6—C10—C9	−0.3 (3)
Fe1—C3—C4—C5	−58.28 (18)	Fe1—C6—C10—C9	−59.47 (19)
C2—C3—C4—Fe1	59.04 (18)	C7—C6—C10—Fe1	59.15 (19)
C3—C4—C5—C1	−1.0 (3)	C8—C9—C10—C6	0.2 (3)
Fe1—C4—C5—C1	−59.71 (17)	Fe1—C9—C10—C6	59.67 (19)
C3—C4—C5—Fe1	58.68 (18)	C8—C9—C10—Fe1	−59.51 (19)
C2—C1—C5—C4	0.9 (3)	C5—C1—C11—C12	10.4 (4)
C11—C1—C5—C4	175.2 (2)	C2—C1—C11—C12	−176.2 (2)
Fe1—C1—C5—C4	60.05 (17)	Fe1—C1—C11—C12	97.8 (3)
C2—C1—C5—Fe1	−59.14 (16)	C1—C11—C12—C13	−177.7 (2)
C11—C1—C5—Fe1	115.2 (3)	C11—C12—C13—O1	176.8 (3)
C10—C6—C7—C8	0.4 (3)		

### 3-Ferrocenylprop-2-en-1-one Hydrogen-bond geometry (Å, º)

*Cg*2 is the centroid of the C6–C10 ring.

**Table:**

*D*—H···*A*	*D*—H	H···*A*	*D*···*A*	*D*—H···*A*
C6—H6···O1^i^	0.95	2.46	3.252 (3)	141
C8—H8···*Cg*2^ii^	0.95	2.82	3.736 (3)	161

Symmetry codes: (i) *x*+1/2, −*y*+3/2, −*z*+1; (ii) *x*+3/2, −*y*−1/2, −*z*.
